# Effect of Green Value Cocreation on Consumer Behavior: A Professional Baseball Franchise’s “Sustainable Party” Event

**DOI:** 10.3390/bs14111050

**Published:** 2024-11-06

**Authors:** Chen-Yueh Chen, Yi-Hsiu Lin, Ming-Ti Shih, Tzu-Yun Yeh

**Affiliations:** 1 Doctoral Program for Transnational Sport Management and Innovation, National Taiwan Sport University, Taoyuan City 333325, Taiwan; chenchenyueh@ntsu.edu.tw; 2 Master Program of Sport Facility and Health Promotion, National Taiwan University, Taipei City 106319, Taiwan; 3 Department of Recreation and Leisure Industry Management, National Taiwan Sport University, Taoyuan City 333325, Taiwan; mingti@ntsu.edu.tw (M.-T.S.); yehtzuyun@ntsu.edu.tw (T.-Y.Y.)

**Keywords:** service-dominant logic, green value cocreation, green purchase intention, team identification, subjective well-being, SMART-PLS statistic

## Abstract

This study examined whether participation in green activities affects the green purchase intention, team identification, and subjective well-being of sports fans through green value cocreation. In addition, this study explored the mediating role of team identification and the moderating role of subjective well-being in green value cocreation. Composite reliability and confirmatory factor analyses were conducted to evaluate the validity and reliability of the research instruments, and structural equation modeling was used to test the study hypotheses. The results indicated that green value cocreation significantly predicted green purchase intention, team identification, and subjective well-being. Team identification partially mediated the relationship between green value cocreation and green purchase intention. However, subjective well-being did not moderate the effect of green value cocreation on green purchase intention. This study investigated how green value cocreation enhances fan participation in environmental actions and translates into green purchase intention. The findings provide insights into the emotional connection between fans and teams and the effect of this connection on green value cocreation and green purchase intention. This study addresses a research gap regarding the role of green value cocreation in professional sports and provides practical insights for businesses, teams, and society to promote environmentally conscious consumption and behavior.

## 1. Introduction

Climate change, ecological degradation, and resource depletion have become common topics in public discourse. These problems affect not only governments and environmental organizations, but also the daily lives of individuals. These challenges can be addressed through sustainable development. However, for sustainable development to be achieved, a balance must be struck to ensure that future generations can maintain similar living standards to those of the present without depleting or degrading the quality of essential resources [1]. The growing prevalence of environmental problems has spurred individual and collective efforts toward promoting sustainability. For example, practices such as recycling, reducing plastic consumption, saving energy, and encouraging renewable energy usage have become widespread social trends. However, resource overconsumption has created major sustainability problems, contributing to global warming, water scarcity, air and soil pollution, and increased waste generation. These sustainability concerns have driven shifts in consumption patterns and behaviors toward environmental sustainability, and this shift has led to green consumption behavior becoming a new paradigm in consumer research [2].

Sports events attract large audiences, with thousands of fans attending in person and numerous others watching games from home. According to the United Nations Framework Convention on Climate Change [3], sports organizations and events can promote sustainable behavior by leveraging fans’ emotional connection to sports [4]. Numerous sports organizations, leagues, and departments have launched environmental initiatives [5]. Many sports teams have started organizing green activities, such as green-themed days, to increase their fans’ environmental awareness and encourage them to participate in sustainability efforts through practical actions. In addition, various sports teams have collaborated with environmental organizations to launch ecofriendly products and initiatives, which has increased fan engagement with environmental problems.

The term “green value cocreation” refers to the process through which consumers and companies engage in exchanges concerning environmental concepts. These exchanges may occur during the production and consumption phases and are initiated to enhance value cocreation [6,7]. The term “green purchase intention” refers to an individual’s willingness to purchase products and services with minimal or no adverse effects on society and the environment. In addition, green purchase intention can be defined as an individual’s inherent desire and intention to purchase environmentally friendly products [8]. Green products are items that do not pollute the planet, do not deplete natural resources, and are recyclable or preservable; such products are typically made from ecofriendly materials or have ecofriendly packaging [9]. Green activities are activities for which sustainable policies or practices have been incorporated into their management and operations [10]. As environmental consciousness has become more prevalent in society, the concept of green value cocreation has increasingly been integrated into the area of sports. Fans are no longer mere spectators, but rather active participants in the cocreation of green value. Businesses operating in environmentally conscious societies have grown to recognize that customers are more inclined to purchase products with ecofriendly characteristics, and this has led to consumers actively engaging in green value cocreation [6]. Participation in green activities can enhance consumers’ willingness to purchase green products, which may in turn positively affect their green consumption behavior [7].

A study reported that sports fans with a strong sense of team identification are more likely to engage in consumption behaviors related to their favorite teams, including purchasing team merchandise [8]. Thus, when sports teams organize green activities, such fans tend to actively participate in and support their favorite team’s environmental initiatives. Such initiatives raise awareness of environmental problems and encourage fans to engage in green consumption behaviors, such as purchasing team-branded ecofriendly products or considering environmental factors when purchasing other products. Economics research has highlighted the importance of understanding consumers’ emotional states to predict their consumption behavior [9]. Consumers’ subjective well-being substantially affects their purchasing decisions [10]. When fans actively participate in green activities organized by their favorite sports teams, they tend to experience a sense of fulfillment that enhances their subjective well-being. This improvement in subjective well-being can translate into emotions that drive actual purchasing behavior.

The positive effects of economic development on living standards have led to public attention becoming increasingly focused on environmental problems [11], resulting in the rise of green consumption behavior as a global trend [12]. The present study examined whether participation in green activities organized by sports teams affects fans’ green purchase intentions through green value cocreation. In addition, this study explored the roles of team identification and subjective well-being in the relationship between green value cocreation and green purchase intention. In particular, this study focused on how green value cocreation encourages fan participation in environmental actions, which in turn fosters green purchase intention. Moreover, this study examined various dimensions of team identification and subjective well-being by analyzing the emotional connections between fans and teams and the effect of these connections on green value cocreation and green purchase intention.

Sports events attract large audiences and have widespread appeal. Such events foster community engagement, strengthen connections between fans and teams, and encourage social media interactions during events. Many sports organizations and event organizers have introduced various environmental sustainability initiatives, such as basic recycling programs, with a focus on reducing carbon emissions. For example, marathon organizers worldwide have started emphasizing the environmental impact of their events [4]. The present study selected the sports environment as its research context and green value cocreation as its focus to determine whether fan participation in green activities affects their green purchase intention, team identification, and subjective well-being through the process of green value cocreation. This study investigated the effects of sustainability initiatives by professional sports teams on spectators’ green purchase intention and their willingness to purchase environmentally sustainable and socially responsible products or services. The present study confirmed the effect of themed events on sports audiences, providing valuable insights for professional sports teams and stakeholders such as sponsors and product suppliers. The findings of this study address a research gap regarding the role of green value cocreation in professional sports and provide practical market insights for businesses, sports teams, and society to promote environmentally conscious consumption and behavior.

## 2. Literature Review

### 2.1. Theoretical Framework

Service-dominant logic (SDL) is a framework in which producers and consumers collaboratively create value through an interactive service delivery process aimed at meeting consumer needs [13]. This framework emphasizes the exchange of applied competencies, enabling individuals and organizations to develop the capabilities required for consumers to achieve their desired level of well-being [14]. The core focus of SDL is value cocreation among all involved parties. Several studies have explored the role of consumers in the value creation process. In traditional value-in-exchange models, companies are positioned as the sole providers of market value, whereas in the SDL model, a value-in-use perspective is applied in which the consumer ultimately determines the value. In this framework, the role of service providers in value creation is diminished to that of offering value propositions instead of being the sole creators of value [15].

In general, value is created through an exchange of resources between parties. However, this process is often imbalanced [16]. Even when resource inequalities exist, the consumer’s perceived value is determined by how effectively resources are integrated, and therefore, consumer assessments of value are unique and personal to the individual. Value cocreation involves multiple participants working to integrate resources to achieve economic or social benefits [17,18].

According to SDL, interactions between consumers and service providers are central to service provision [19]. Expertise, technical capabilities, and interactive experiences affect the service provision process. These interactions enable consumers and service providers to develop mutual understanding, build trust, learn from each other, grow, and cocreate value. Thus, SDL can be considered a feasible alternative to goods-dominant logic. Sports activities offer platforms for fans and spectators to participate in value cocreation [20], and in the SDL framework, value is cocreated through the integration of resources and interactions among stakeholders.

**Figure 1:**
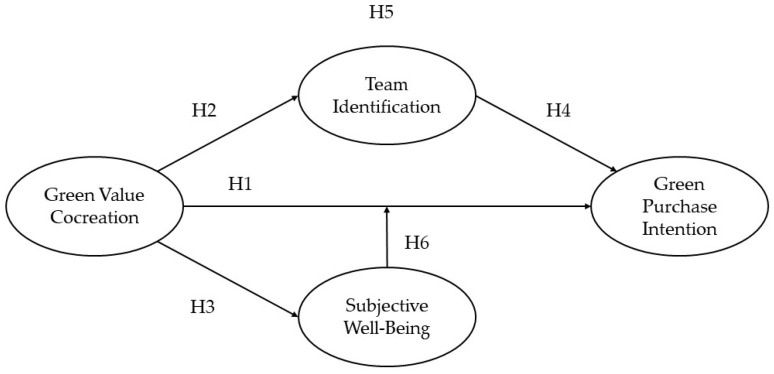
Research framework. Note: H1–H4 are direct effects, H5 is a mediating effect, and H6 is a moderating effect.

This study examined a professional baseball franchise’s Sustainable Party event as a platform for value cocreation between fans and their sports teams. The study investigated how green value cocreation that arises from interactions between fans and their favorite sports teams affects fans’ green purchase intention, team identification, and subjective well-being (Figure 1).

### 2.2. Green Value Cocreation

Cocreation refers to the generation of new value through collaboration. This concept has been widely explored and applied in service management, innovation management, marketing, and consumer research [21]. In value creation, service providers must not only deliver value but also explain the value of their products to consumers. Consumers then integrate the service provider’s products with those offered by other market participants (e.g., services from other providers) and the public domain (e.g., public infrastructure) and with their personal resources (e.g., knowledge and skills) to generate value. Therefore, the effectiveness of value creation mainly depends on how efficiently consumers integrate and use available resources [22,23].

The concept of value cocreation can be divided into two main aspects: coproduction and value in use. Coproduction involves consumers actively participating in the designing of products and services by sharing information or knowledge or by assuming specific roles. By contrast, value in use involves consumers’ evaluations of a product or service on the basis of their personal experience with it [24,25].

The primary goal of value cocreation is to generate new value through cooperation. In the context of sports, value in use involves all components of a sports event, including fans, teams, players, venues, weather, and competition, with these elements being interrelated and shaping the overall sports experience. Coaches, referees, and venue staff also play crucial roles in value cocreation [26].

Societal concerns regarding environmental and sustainability problems have increased, and therefore, incorporating environmental requirements into value cocreation has become a key aspect of achieving sustainability goals. The concept of green value cocreation, which was first introduced by Chang [27], refers to the process through which companies and their partners share environmental concepts, creating value through participation in either the production or consumption stage [28]. Green value cocreation involves a collaborative effort between consumers and companies aimed at increasing environmental awareness. This can occur during both the production stage and the consumption stage [29,30]. In the current business environment, companies recognize that consumers have become increasingly concerned about ecological protection and are more likely to purchase environmentally friendly products. Therefore, companies are more actively promoting green value cocreation [6].

Corporate social responsibility (CSR) is a broad concept that encompasses various areas, including corporate governance, legal compliance, community involvement, employee rights, environmental suitability, philanthropy, market relations, and other welfare practices. By implementing CSR policies, companies and organizations self-regulate and integrate sustainability into their daily operations [31]. Green value cocreation that involves the exchange of environmental concepts between consumers and companies [29,30] can therefore be considered an extension or a goal of CSR. The present study focused on the concept of green value cocreation, a topic that has received limited attention in the literature. Because CSR involves corporate responsibilities toward society and the environment, the theoretical framework of the concept can provide a foundation for developing hypotheses related to green value cocreation.

This study focused on the Rakuten Monkeys’ annual Sustainable Party event as its primary research subject. The main goal of this event, which is cohosted by the Taoyuan City Government and the Rakuten Monkeys, is to transform the team’s home stadium into the nation’s first sustainable baseball stadium, with low-carbon emissions and zero waste. The event has a focus on environmental and safety topics and is advertised for using promotional materials that can be recycled and repurposed after the event. In addition, the event involves various green value cocreation activities. For example, attendees are encouraged to use reusable cups; discounts are provided to those who bring their own water bottles; and fans who wear ecofriendly jerseys, bring reusable cups or towels, or present proof of having used public transportation on the day of the event receive limited-edition gifts. In addition, Global Mall Taoyuan A19 constructs a temporary ecofriendly amusement park and organizes an online treasure hunt called “Sustainable Adventurer”, in which fans could complete tasks to have their names entered into a raffle for hotel vouchers [32].

Cocreation activities not only provide organizations with opportunities to inform fans of the organizations’ environmental initiatives, but also enhance fans’ environmental awareness as they actively participate in such initiatives. The current study applied the concept of green value cocreation to professional sports events, focusing on the importance of environmental collaboration between sports teams and their audiences in jointly creating a more sustainable sports environment.

### 2.3. Green Value Cocreation and Green Purchase Intention

Berrone, et al. [33] categorized corporate green marketing into substantive and symbolic activities. Substantive activities involve major business changes, such as introducing new environmental technologies, improving production techniques, developing ecofriendly products and services, and increasing waste management efficiency. Symbolic activities involve using a company’s environmental image to gain consumer recognition without altering core business operations. This can be achieved by promoting environmental awareness, establishing environmental committees, applying for green certifications, and participating in environmental initiatives. Corporate green marketing positively affects consumers’ green purchase intention. Notably, compared with symbolic activities, substantive green marketing activities are more effective forms of corporate green marketing [34].

Consumers who actively engage in value cocreation are more likely to purchase green products or services [35]. Research indicates that, when consumers cocreate value with companies, their purchase intentions increase [36]. CSR activities may positively affect consumers’ green purchase intention [31]. Furthermore, a study reported that virtual CSR cocreation enhances consumers’ intentions to purchase green products, and higher experiential value in virtual CSR cocreation results in stronger green purchase intention [7].

In summary, sports teams can enhance fan purchase intentions through value cocreation activities. Therefore, this study hypothesized that participation in green activities organized by sports teams affects fans’ green purchase intentions through green value cocreation. The hypothesis is as follows:

**H1:**  *Green value cocreation significantly predicts green purchase intention.*

### 2.4. Green Value Cocreation and Team Identification

During value cocreation, team brand identification has a strong influence on fans, audiences, and other stakeholders, such as sponsors [37]. However, building team brand identification requires a solid foundation. Thus, team brand cocreation must be effectively managed. Sports brand managers can transform their brand into a platform for customer engagement, which can foster interactions between consumers (e.g., audiences and sponsors) and fan communities. According to marketing theory, products are continually cocreated through interactions between consumers and organizations. In the sports context, these interactions often involve ritualistic behaviors performed before, during, and after professional sports events [38]. Fans who participate in these ritualistic behaviors tend to develop a strong sense of identification with their team.

Value cocreation exerts a strong positive effect on fan identification [39]. When sports teams offer fans opportunities to engage in cocreation activities, fan identification with the team strengthens [40]. Furthermore, in the sports domain, audiences who perceive an organization as being committed to CSR develop a greater sense of pride in and identification with the team [41]. In other words, when sports organizations actively promote CSR, they foster customer pride, which contributes to stronger team identification.

In summary, increasing interactions between fans and sports teams enhances fan identification. Therefore, the present study hypothesized that sports teams that actively address social and environmental problems through green value cocreation lead their fans to recognize the team’s commitment to addressing these problems, thereby strengthening their identification with the team. The hypothesis is as follows:

**H2:**  *Green value cocreation significantly predicts team identification.*

### 2.5. Green Value Cocreation and Subjective Well-Being

In the context of leisure activities, individuals who participate in such activities who engage in value cocreation tend to experience high levels of satisfaction and subjective well-being [42]. Personal environmental behaviors, such as ecological consumption and recycling, are positively correlated with subjective well-being [43]. Engaging in value cocreation activities not only enhances individual well-being, but also positively affects other customers, organizations, and society [44].

When customers participate in value cocreation, the emotional and cognitive satisfaction they experience substantially enhance their subjective well-being [45]. Furthermore, higher perceived CSR is associated with greater subjective well-being. Thus, companies should prioritize fulfilling their social responsibilities to enhance their reputation and maintain a positive image both internally and externally [46].

In summary, value cocreation and environmental behaviors positively affect subjective well-being. Therefore, this study hypothesized that, in the context of sustainable development, value cocreation activities promote active interactions among sports teams, sponsors, and fans. Engaging in environmentally conscious behaviors leads fans to feel that they are contributing to the preservation of the environment, which enhances their self-satisfaction and well-being. The hypothesis is as follows:

**H3:**  *Green value cocreation significantly predicts subjective well-being*.

### 2.6. Green Purchase Intention

The term “intention” refers to conscious planning and willingness to engage in a specific behavior [47]. The term “purchase intention” refers to a consumer’s tendency toward and preference for purchasing a specific product or service, with such intention involving evaluating options and making a purchase decision. Various factors affect purchase intention, and a consumer’s final selection of a product or service is determined by key external factors that influence such an intention [48,49]. Specifically, purchase intention involves an individual making a conscious plan and being willing to purchase a specific brand or product [50]. This concept has been widely applied in the field of marketing, and numerous companies have used it to predict sales rates of new products and repurchase rates of existing products [51].

Purchase intention and green purchase intention are both related to individual attitudes toward a product or brand, but they differ in key aspects. Green purchase intention refers to the extent to which an individual is willing to consider and to which they prefer environmentally friendly products over traditional alternatives when making purchasing decisions [52,53,54]. Green purchase intention can also be defined as the desire or willingness to purchase products that have minimal environmental impact and are environmentally friendly. Individuals with high green purchase intention are more likely to purchase ecofriendly products, even when they are more expensive than traditional options [55].

The present study explored how the degree of green purchase intention among sports fans changes after their participation in green value cocreation.

### 2.7. Team Identification

The term “team identification” refers to the extent to which fans form a psychological connection with their favorite sports team [8]. This concept originates from social identity theory [56], which explores how individuals form a sense of belonging and identification with social groups based on their perceptions of those groups. According to social identity theory, individuals tend to compare and differentiate the values and norms of their own group from those of other groups. They typically exhibit positive attitudes toward the values and norms of their own group and bias against those of other groups [57].

According to social identity theory, consumer identification with a sports team, that is, team identification, forms part of an individual’s self-concept, with the team being perceived as a central component of community membership. Such identification develops because fans derive emotional value from belonging to a group, as well as from their community knowledge, involvement, and evaluation [58]. Studies on sports marketing have examined team identification in various contexts, revealing it to be associated with several emotional, cognitive, and behavioral outcomes. For example, fans with a strong sense of team identification tend to exhibit strong negative reactions when their team loses and strong positive reactions when their team wins, whereas fans with a weak sense of team identification tend not to experience strong emotional responses after a game [59]. Compared with fans with a weak sense of team identification, those with a strong sense of team identification are more likely to exhibit favoritism toward their team [60].

The present study examined the relationship between team identification and green purchase intention and explored the potential mediating role of team identification in the relationship between green value cocreation and green purchase intention.

### 2.8. Team Identification and Green Purchase Intention

Fans with a stronger sense of team identification are more likely to purchase team-related merchandise [61] and engage in more proactive support behaviors, such as frequently attending games and purchasing team-related products [62]. Team identification positively affects purchase intention. Fans with a stronger sense of team identification are more likely to purchase from brands recommended by sports influencers they follow on social media [8], and having a strong sense of identification with a sports team enhances positive attitudes toward that team and strongly predicts consumer behavior [63].

Strengthening the emotional connection between fans and their sports teams can enhance fans’ purchase intention and drive sales. In the context of sustainability, team identification may encourage fans to participate in activities that support environmental initiatives promoted by their team, which, in turn, can affect their intention to purchase green products. On the basis of these concepts, this study proposes the following hypothesis:

**H4:**  *Team identification significantly predicts green purchase intention*.

### 2.9. Team Identification, Green Value Cocreation, and Green Purchase Intention

Zhang and Ahmad [64] reported significant correlations among CSR, brand image, consumer–company identification, and identity attractiveness, all of which enhance consumer satisfaction and purchase intention. Consumers tend to buy products from companies that prioritize CSR and actively support CSR activities. Team identification strengthens the relationship between fans’ valuation of CSR and their patronage intentions. In addition, fans with a stronger sense of team identification tend to engage more frequently in direct viewing behaviors and have higher consumption of team-related products [58].

Consumer identification with an organization’s CSR activities, brand image, and brand attachment can positively affect purchase intention. Thus, companies should encourage consumers to engage with their CSR initiatives [65]. Perceived CSR is positively correlated with consumer satisfaction and team identification, both of which affect the consumption behavior of sports fans [66]. Participation in CSR activities also affects consumers’ self-construal and green product purchase intentions, and virtual community identification plays a mediating role in this process [7].

In summary, team identification plays a critical role in the relationship between CSR and purchase intention. CSR activities should involve consumer participation to strengthen consumers’ emotional connection with and trust in companies. By contrast, value cocreation focuses on interactions between customers and companies. This study hypothesized that team identification mediates the relationship between green value cocreation and green purchase intention. By actively engaging with social and environmental problems, sports teams can strengthen their fans’ identification with the team, encouraging fans to participate in green activities or team-organized initiatives and ultimately influencing their green purchase intention. The hypothesis is as follows:

**H5:**  *Team identification mediates the relationship between green value cocreation and green purchase intention.*

### 2.10. Subjective Well-Being

In 1948, the World Health Organization defined health as both the absence of disease and the presence of well-being and happiness. Happiness is affected by various factors, with positive emotional responses being the most prominent. Argyle [67] reported that the key components of happiness include the frequency and intensity of positive emotional experiences, one’s overall level of life satisfaction, and the absence of negative psychological conditions, such as depression and anxiety.

Subjective well-being refers to an individual’s emotional responses and overall life satisfaction [68]. Self-reported measures are typically used to evaluate life satisfaction, happiness, and subjective well-being [69]. Diener, Suh, Lucas and Smith [68] identified seven major life domains that affect subjective well-being: health, financial status, self-perception, family, social relationships, work, and leisure. Research on psychology, sociology, economics, entertainment, and tourism indicates that self-evaluated health, financial status, self-worth, marital status, social relationships, job satisfaction, and leisure are positively correlated with subjective well-being [70].

Consumer behavior theory, which was first proposed by Duesenberry [71], posits that various forms of consumption can enhance well-being in at least three manners, thereby improving subjective well-being. Spending money on leisure or charitable activities can increase happiness by improving social relationships [72]. A consumer’s emotional state is a key predictor of their behavior [9]. When brands communicate environmental CSR messages, these messages positively affect consumers’ attitudes toward the brand, which in turn enhances their purchase intention and willingness to recommend the brand. CSR messages not only affect consumers’ purchase behaviors but also encourage sustainable actions, contributing to enhanced subjective well-being [73].

In summary, consumer behavior and happiness are closely connected. Consumption behaviors, such as spending money on charitable activities, can enhance consumers’ subjective well-being, and value cocreation can increase consumers’ purchase intention of various products or services. This study hypothesized that participation in green value cocreation activities would enhance sports fans’ subjective well-being, which would in turn enhance their willingness to purchase green products. In addition, this study hypothesized that fans with higher subjective well-being might be more likely to exhibit socially expected behaviors, such as green purchasing. The hypothesis is as follows:

**H6:**  *Subjective well-being moderates the relationship between green value cocreation and green purchase intention.*

## 3. Materials and Methods

### 3.1. Research Setting

The Rakuten Monkeys is a professional baseball team in Taiwan that competes in the Chinese Professional Baseball League (CPBL), which is currently Taiwan’s only baseball league. The CPBL consists of six teams: the Wei Chuan Dragons, the CTBC Brothers, the Uni-President 7-Eleven Lions, the Rakuten Monkeys, the TSG Hawks, and the Fubon Guardians. The Rakuten Monkeys were selected for the present study because they have hosted sustainability-themed events for two consecutive seasons and reached the Taiwan Series (the CPBL championship) in the most recent baseball season.

This study focused on the Rakuten Monkeys’ Sustainable Party event that was held on 27 and 28 April 2024 to celebrate Earth Day. This event was jointly organized by the Taoyuan City Government and the Rakuten Monkeys and focused on addressing environmental problems. The goal of the event was to transform the Rakuten Monkeys’ home stadium into the nation’s first low-carbon, zero-waste baseball stadium. Ecofriendly decorations and materials, such as recyclable event banners that could be repurposed into new products, were used throughout the event. The Sustainability Party included green value cocreation activities. Fans were encouraged to use reusable cups, and those who brought their own water bottles received beverage discounts. Limited-edition stadium gifts were offered to fans who wore ecofriendly jerseys, brought reusable cups or towels, or provided proof of having used public transportation on the days of the event. In addition, the Global Mall Taoyuan A19 established a temporary environmental, social, and governance–themed amusement park and organized an online orienteering game named “Sustainable Adventurer”, where participants who completed specific tasks could enter a drawing to win hotel vouchers [32]. These cocreation activities enabled fans to participate in environmental initiatives and enhance their environmental awareness through participation and action.

### 3.2. Participants

This study was approved by the Institutional Review Board of National Taiwan Sport University. Event attendees who purchased tickets to the Rakuten Monkeys’ Sustainable Party were invited to participate. Participants were recruited at the Sustainability Party, which was held in the infield section of the stadium. Because spectators with outfield tickets could not access the event area, individuals who did not attend the event in person and those with outfield tickets were excluded. In total, 128 participants, including 75 (58.59%) men and 53 (41.41%) women, were recruited.

### 3.3. Research Instrument

The following scales were used: the Green Value Cocreation Scale (five items adapted from Borah, et al. [74]), the Green Purchase Intention Scale (four items adapted from Mahmood, Siddiqui and Tahir [55]), the Team Identification Scale (five items adapted from Lee [8]), and the Subjective Well-Being Scale (six items adapted from Mathis [42]). All responses were rated on a five-point Likert scale, with endpoints ranging from 1 (strongly disagree) to 5 (strongly agree). Back-translation was used to ensure that the translated scales accurately reflected the meaning of the content in the original measurement instruments.

Scholars indicate that composite reliability (CR) values should be greater than 0.6 [75]. In the present study, all CR values exceeded 0.9, indicating high internal consistency of the research tools. Validity was evaluated using confirmatory factor analysis. Research has indicated that standardized factor loading values should be greater than 0.5 [76] and average variance extracted (AVE) values should be greater than 0.5 [75]. In the present study, standardized factor loading values ranged from 0.68 to 0.93, and all AVE values were greater than 0.7, indicating the strong convergent validity of the research tools (Table 1). Aligning with the recommendations of Fornell and Larcker [75] and Gaski and Nevin [77], the correlation coefficients between all construct pairs were lower than the square root of the AVE values for each construct, indicating satisfactory discriminant validity (Table 2).

To address the problem of common method variance, the questionnaire items were carefully worded to prevent ambiguity [78]. In addition, Harman’s single-factor test (exploratory factor analysis), the Kaiser–Meyer–Olkin test (0.93), and Bartlett’s test of sphericity (χ^2^ = 2420.17, degrees of freedom = 190, *p* < 0.001) were conducted. The results met the recommended thresholds for factor analysis [79]. However, the first factor explained 55.47% of the variance. In consideration of this fact, future studies on similar topics should examine the potential effect of common method bias.

The present study used the variance inflation factor (VIF) to examine multicollinearity. The results indicated an absence of multicollinearity, because all VIF values were below the threshold of five (green value cocreation = 1.48, team identification = 1.73, and subjective well-being = 2.12) [80].

### 3.4. Data Processing and Analysis

Structural equation modeling (SEM) was performed to examine the relationships between the latent variables and to measure the strength of those relationships. Bootstrapping with 2000 repetitions at a 95% confidence level was applied to analyze mediating effects [81]; a bias-corrected percentile method was used for interval estimation. A multigroup analysis was performed to examine moderating effects, and a cluster analysis was conducted to identify relevant clusters. A chi-square difference greater than 3.84 with an increase of one degree of freedom indicated a significant difference between groups after constraints were imposed [82]. The critical value of a chi-square distribution with one degree of freedom at a significance level of 0.05 is 3.84. Statistical analysis was performed using SPSS 20.0, whereas SEM and analyses of mediating and moderating effects were conducted using AMOS 25.

## 4. Results

### 4.1. Path Analysis

**Figure 2:**
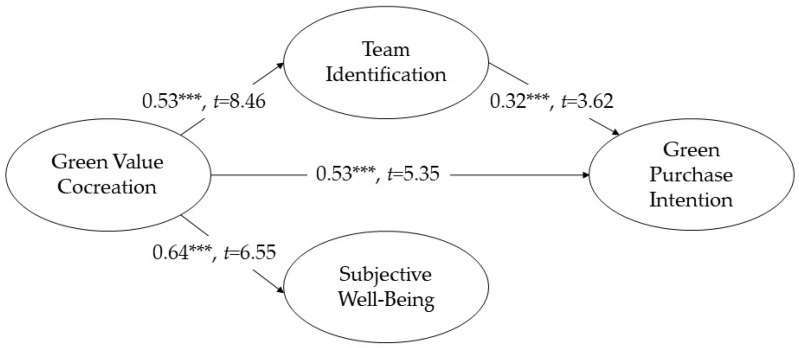
Path Analysis Diagram Note: *** indicates *p* < 0.001; β = standardized path coefficient; the results of the mediating effect analysis are presented in Table 3; the results of the moderating effect analysis are presented in Figure 3.

Figure 2 presents the SEM results. Green value cocreation was positively correlated with green purchase intention (β = 0.53 ***, *t* = 5.46), which supports H1. In addition, green value cocreation was positively correlated with team identification (β = 0.53 ***, *t* = 5.35), which supports H2. Green value cocreation was also positively correlated with subjective well-being (β = 0.64 ***, *t* = 6.55), which supports H3. Team identification was positively correlated with green purchase intention (β = 0.32 ***, *t* = 3.62), which supports H4. The coefficients ranged from 0.76 to 0.84 for green value cocreation, from 0.80 to 0.93 for green purchase intention, from 0.67 to 0.92 for team identification, and from 0.79 to 0.93 for subjective well-being. All items were statistically significant.

### 4.2. Mediating Effect Analysis

The bootstrapping results revealed that the total standardized effect estimate was 0.70, with a bias-corrected *p*-value of <0.05 and a confidence interval that excluded 0, indicating a potential mediating effect. The indirect standardized effect estimate was 0.17, with a bias-corrected *p*-value of <0.05 and a confidence interval that excluded 0, indicating the presence of a mediating effect. The direct effect estimate was 0.53, with a bias-corrected *p*-value of <0.05 and a confidence interval that excluded 0, indicating the presence of a partial mediating effect (Table 3).

### 4.3. Moderating Effect Analysis

**Figure 3:**
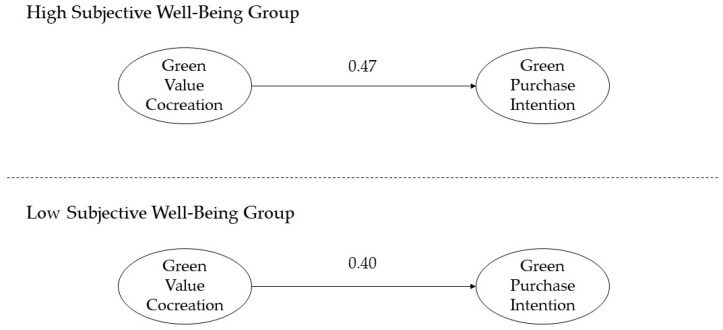
Moderating effect of subjective well-being on the relationship between green value cocreation and green purchase intention. Note: Degree of freedom = 1; χ^2^ = 264.295 − 263.625 = 0.67.

Cluster analysis was conducted to categorize participants’ subjective well-being as high (M = 4.32, *n* = 101) or low (M = 2.85, *n* = 27). Subsequently, a multigroup analysis was conducted. The results revealed a chi-square difference of 0.67, which is less than the threshold of 3.84. Therefore, H6 was rejected (Figure 3).

## 5. Discussion

### 5.1. Green Value Cocreation Positively Predicts Green Purchase Intention

The results of the present study revealed that green value cocreation significantly predicts green purchase intention, which is consistent with the findings of other studies. Multiple e-commerce studies have indicated the importance of the relationship between value cocreation and purchase intention, emphasizing that consumer participation in the cocreation of products or services can enhance consumer intent to purchase products or services [83]. Consumers actively involved in value cocreation exhibit increased purchase intentions [84], and individuals who engage in the cocreation process develop a strong connection with the final product and are more likely to purchase it [85]. A study on agricultural products in China determined that CSR activities encourage consumers to participate in value cocreation, which subsequently affects their green purchase behaviors [86]. Another study reported that CSR activities positively affect consumers’ green purchase intentions in the Chinese market [31].

In the current study, attendees of the Rakuten Monkeys’ Sustainable Party reported feeling that their participation in value cocreation contributed to environmental protection. In addition, the event increased their environmental awareness and sense of social responsibility, which in turn motivated them to purchase ecofriendly products. These findings indicate that participating in green value cocreation not only enhances consumers’ environmental consciousness but also encourages them to change their consumption habits. SDL emphasizes the importance of collaboration and interaction in value cocreation. In the present study, interactions between fans and the team fostered a sense of personal contribution to environmental protection and social responsibility, which ultimately affected fans’ consumption behavior.

### 5.2. Green Value Cocreation Positively Predicts Team Identification

The current study revealed that green value cocreation significantly predicts team identification. This finding is consistent with those of previous studies. In the sports domain, identification is a key factor that should be considered in analyses of cocreated value [37], and value cocreation exerts a positive and significant effect on fan identification [39]. A study on CSR in sports revealed that team identification partially explained the relationships between high affective evaluation and high merchandise consumption, word of mouth, and repeat purchases. Fans who perceived their team as exhibiting a strong sense of CSR felt a deeper connection to the team [58]. Another study suggested that CSR activities in the sports industry can enhance team identification and emotional attachment. Such activities can thus be employed as a marketing strategy by sports teams and organizations to increase team identification [66].

By participating in team-organized activities, fans create emotional value, which further strengthens their identification with the team. This concept is consistent with the principle of SDL. In the current study, individuals who attended the event reported a sense of having a shared goal with the team and considered themselves to be part of the team through their involvement in value cocreation. This experience enhanced their sense of belonging and emotional connection with the team, indicating that green value cocreation fosters emotional bonds between fans and sports teams, which in turn strengthens team identification.

### 5.3. Green Value Cocreation Positively Predicts Subjective Well-Being

The present study revealed that green value cocreation significantly predicts subjective well-being. This finding is consistent with those of previous studies. A study on travel experiences reported that cocreation has a positive effect on well-being [87]. In addition, a study on educational management indicated that cocreation activities can enhance students’ well-being by fostering collaboration [88]. Furthermore, cocreation experiences can predict consumer well-being [89]. A study indicated that participants interacting with others during the cocreation process experienced higher service value and improved subjective well-being [90]. Another study found that value cocreation provided participants with moral experiences and greater civic awareness, enhancing their well-being and causing them to perceive themselves as having made a positive societal contribution [91].

In this study, individuals who attended the Sustainable Party event reported feeling that their participation in value cocreation contributed to environmental protection and increased their awareness and concern for environmental problems. This experience enhanced their subjective well-being by helping them recognize their personal value. Participants also reported feeling more socially involved because of their participation in cocreation activities, which improved their subjective well-being. This finding is consistent with the principles of SDL, because interactions between fans and the team they supported during green activities fostered the cocreation of environmental value, which in turn enhanced the fans’ subjective well-being.

### 5.4. Team Identification Positively Predicts Green Purchase Intention

The present study revealed that team identification significantly predicts green purchase intention, which is consistent with findings in the literature. A study on green food products indicated that, when consumers identify with a group or ideology, their purchase intentions increase [92]. Another study reported that social identity affects fans’ willingness to purchase green products [93]. Research on green consumer behavior indicates that social identity affects consumers’ willingness to purchase green products [94]. In addition, team image positively affects both team identification and purchase intention, with a more positive team image increasing both team identification and purchase intention [95]. Furthermore, stronger team identification significantly predicts higher purchase intention, because fans with stronger team identification are more likely to purchase team-branded products [96].

The results of the present study indicate that fans support their team’s green initiatives because of their sense of team identification, which increases their intention to purchase green products. They also tend to purchase green products to gain social or group approval by indicating that they share values with their favorite team.

### 5.5. Team Identification Partially Mediates the Relationship Between Green Value Cocreation and Green Purchase Intention

The present study revealed that team identification partially mediates the relationship between green value cocreation and green purchase intention. This finding is consistent with those of previous studies. A study on CSR in the sports domain reported that team identification partially explained the relationships between high affective evaluation and increased merchandise consumption, word of mouth, and repeat purchase intention. When fans are satisfied with their favorite team’s CSR performance, their emotional connection with the team strengthens, resulting in more merchandise purchases and active participation in events [58]. Social value cocreation can enhance consumers’ purchase intention through consumer–company identification [97]. In addition, team identification moderates the relationship between CSR activities and purchase intention, with fans who exhibit a stronger sense of team identification being more affected by CSR efforts and demonstrating greater support for their team [98].

Most relevant studies have investigated the effect of CSR on team identification and consumer behavior. By contrast, the current study examined how green value cocreation affects purchase intention through the emotional connection between fans and their favorite sports team. Although both CSR and green value cocreation activities emphasize CSR, the focus of green value cocreation is more on the interactive cocreation of environmental value between companies and consumers. The results of the current study indicate that green value cocreation activities strengthen the emotional bond between fans and their favorite sports team, increasing fans’ willingness to support the team’s green initiatives and modify their consumption habits to include green products. After participating in green value cocreation activities, fans may become more likely to purchase environmental products to demonstrate that they share values with their team and to apply the environmental knowledge they have gained.

### 5.6. Subjective Well-Being Does Not Moderate the Relationship Between Green Value Cocreation and Green Purchase Intention

The current study revealed that subjective well-being does not moderate the relationship between green value cocreation and green purchase intention. This result is in agreement with those of several studies. For example, Ahmed, et al. [99] suggested that CSR, employee well-being, and environmental awareness positively affect green behaviors. However, in the present study, the moderating effect of subjective well-being was nonsignificant. This discrepancy may be attributed to the nature of the green activities examined in the present study, which were short-term events. Emotions generated by short-term activities are often transient, and the limited scale or depth of such events may be insufficient to significantly affect future purchasing decisions, even if fans experience an increase in their subjective well-being after participating in value cocreation activities. In addition, green value cocreation activities may directly affect fans’ green purchase intentions, independent of any moderating effect of subjective well-being.

## 6. Conclusions

The present study applied the perspective of SDL theory, which focuses on the interaction between consumers and service providers [19], thereby deepening the understanding of green value cocreation and its crucial role in promoting sustainable development through sports. Sustainability is not solely the responsibility of sports teams, and the results of this study indicate that fans who participate in green activities organized by their favorite sports teams are more likely to cocreate value related to environmental awareness, social responsibility, and well-being. This finding is consistent with the emphasis of SDL on the cocreation of new value through collaboration [20,100,101]. Moreover, this study expanded the application of green value cocreation to the professional sports domain. Previous studies have rarely explored green value cocreation in sports events. The present study fills this gap by demonstrating that sustainability activities can strengthen the emotional bond between spectators and sports teams, thereby enhancing fans’ team identification and green purchase intentions.

The main objective of green value cocreation is to generate new value through cooperation [26]. The current study revealed that participation in cocreation activities increases fans’ identification with their favorite team, which in turn enhances the emotional bond between fans and the team. Thus, fans become more willing to support the team’s green initiatives and adjust their consumption habits to include more green products. Team identification partially mediates the relationship between green value cocreation and green purchase intention. Fans may experience a strong sense of self-worth and feel that they have contributed to environmental efforts after participating in green value cocreation activities, which can increase their subjective well-being. However, although cocreation activities enhance subjective well-being, they may directly affect fans’ green purchase intentions. Thus, the moderating effect of subjective well-being in this context is nonsignificant.

The findings of this study offer several practical implications. For example, they indicate that professional sports teams can organize more sustainability-related events or provide sustainability-related knowledge to help fans better understand sustainable practices through participation in sports events. Such engagement may encourage fans to use more ecofriendly products in their daily lives. In addition, participation in these events can enhance fans’ subjective well-being, making them feel happier after attending professional sports games and enriching their overall experience, which may in turn increase their willingness to attend future events. Sports teams can also collaborate with stakeholders (e.g., sponsors and suppliers) to promote sustainability through joint activities while introducing fans to sponsors’ products. Such collaboration can enhance the brand images of both teams and their sponsors, improving fans’ perceptions of and attitudes toward them.

The current study identified team identification as a key factor. By hosting sustainability activities, professional sports teams can demonstrate their commitment to sustainable development, thereby enhancing fans’ identification with the team. The current study’s discovery of a mediating effect of team identification indicates that green value cocreation activities increase fans’ sense of identification with their team, which in turn positively affects their green purchase intentions. This highlights the strong connection between fans and teams. In summary, the impact of sustainability-related activities organized by professional sports teams on fans should not be underestimated. These activities not only help attract more fans but also contribute to the achievement of sustainability goals.

### Research Limitations and Directions for Future Studies

The present study had several limitations. This study focused on the Rakuten Monkeys’ Sustainable Party. The original intention of this study was to distribute electronic questionnaires on site to ensure that participants were actual attendees. However, because of unstable weather and the smaller fan base of the Rakuten Monkeys than that of other teams, actual attendance was considerably lower than expected. Moreover, Sustainable Party events are usually organized to coincide with Earth Day and are not repeated. Future research should consider examining similar events organized by other teams or across various sports to enhance the data reliability. Such an approach may facilitate the collection of diverse data for comparisons across different matchups or sports. Furthermore, the limited sample size rendered multigroup invariance testing between participants with high versus low subjective well-being challenging. Future studies should address this problem to ensure more robust findings. The concept of sustainable well-being has become increasingly important [102]. Future research may consider this concept to further enhance the understanding of well-being in the context of sustainability.

This study focused on sustainability-themed events organized by professional sports organizations. However, these are one-off events rather than ongoing activities. Future studies should investigate the impact of long-term sustainability initiatives on consumers’ green purchase intentions over time. Furthermore, this study focused on professional baseball; future research could expand its focus to other sports, considering both large-scale events (e.g., the Olympics) and individual sports (e.g., marathons), to determine whether green value cocreation processes in other sports also predict green purchase intentions. This study determined that team identification partially mediated the relationship between green value cocreation and green purchase intentions. Future studies could identify other potential mediating variables. In addition, subjective well-being did not significantly moderate the relationship between green value cocreation and green purchase intentions in this study. Future research could consider other potential moderators or expand investigation to other domains to assess the generalizability of the current findings. Finally, the problem of common method variance identified in this study warrants further investigation in future research.

## Figures and Tables

**Table 1:** Confirmatory Factor Analysis Results (N = 128).

Variable/Item	M	SD	λ	*t*	CR	AVE
Green Value Cocreation (GVCC)					0.92	0.70
1. I believe that the Rakuten Monkeys actively share relevant ideas and measures with their fans when implementing green activities. (GVCC1)	4.05	0.91	0.85	11.74		
2. I believe that the Rakuten Monkeys are willing to invest time and effort into collaborating with their fans to improve their green activities and achieve more favorable environmental and sustainable development outcomes. (GVCC 2)	4.13	0.94	0.85	11.70		
3. I believe that fans can easily access information regarding the Rakuten Monkeys’ environmental efforts. (GVCC 3)	3.97	0.94	0.75	9.81		
4. I believe that the green activities organized by the Rakuten Monkeys meet fans’ needs and expectations regarding environmental protection. (GVCC 4)	4.02	0.97	0.90	12.84		
5. I believe that fan participation and support are crucial to the success of the Rakuten Monkeys’ green activities, enabling the team and fans to achieve environmental protection and sustainable development goals. (GVCC 5)	4.20	0.84	0.83	11.24		
Green Purchase Intention (GPI)					0.93	0.76
1. After attending the Sustainability Party, I started considering purchasing products with minimal environmental impact. (GPI 1)	4.09	0.87	0.90	12.97		
2. After attending the Sustainability Party, I started considering choosing products from green brands to protect the environment. (GPI 2)	4.12	0.84	0.93	13.62		
3. After attending the Sustainability Party, I started considering using ecofriendly products. (GPI 3)	4.05	0.82	0.85	11.87		
4. After attending the Sustainability Party, I started considering purchasing green products. (GPI 4)	4.00	0.88	0.79	10.59		
Team Identification (TI)					0.92	0.70
1. I am interested in others’ opinions regarding the Rakuten Monkeys. (TI 1)	4.10	0.88	0.68	8.52		
2. When someone praises the Rakuten Monkeys, I feel as if I am being personally praised. (TI 2)	4.03	1.06	0.87	12.10		
3. When I talk about the Rakuten Monkeys, I usually use “we” instead of “they”. (TI 3)	4.09	1.13	0.88	12.35		
4. The success of the Rakuten Monkeys feels like my success. (TI 4)	3.95	1.17	0.92	13.40		
5. When someone criticizes the Rakuten Monkeys, I feel as if I am being personally insulted. (TI 5)	3.59	1.17	0.82	11.05		
Subjective Well-Being (SWB)					0.95	0.76
1. During the Sustainability Party, I achieved many environmental goals, and this experience enriched me in many aspects. (SWB 1)	3.98	0.85	0.81	10.91		
2. Attending the Sustainability Party was rewarding in many aspects; I feel more satisfied with myself and various aspects of life after this green event. (SWB 2)	3.98	0.84	0.86	12.18		
3. Overall, I feel that the Sustainability Party enriched my life, and I am glad that I participated in it. (SWB 3)	4.09	0.86	0.89	12.68		
4. After attending the Sustainability Party, my life satisfaction increased. (SWB 4)	3.96	0.93	0.90	12.96		
5. Overall, the experience of participating in this green event was memorable and improved my quality of life. (SWB 5)	3.92	0.99	0.92	13.58		
6. Overall, I felt very happy after attending this green event. (SWB 6)	4.13	0.77	0.83	11.39		

Note: χ^2^ = 311.49; degrees of freedom = 164; χ^2^/degrees of freedom = 1.90; *p* < 0.001; goodness-of-fit index = 0.81; nonnormed fit index = 0.93; comparative fit index = 0.94; root mean square error of approximation = 0.08; standardized root mean square residual = 0.06. Abbreviations: M, mean; SD, standard deviation; λ, factor loading; *t*, *t*-value.

**Table 2:** Discriminant Validity Analysis Results.

	GVCC	GPI	TI	SWB
GVCC	0.84			
GPI	0.67	0.87		
TI	0.51	0.60	0.84	
SWB	0.61	0.73	0.65	0.87

Note: Diagonal values are the square roots of AVE values for each latent variable. These values should be greater than nondiagonal values. Abbreviations: GVCC, green value cocreation; GPI, green purchase intention; TI, team identification; SWB, subjective well-being.

**Table 3:** Mediating Effect Analysis Results.

	Standardized Effect	95% Confidence Interval	
		BC *p*-Value	BC
Total Effect			
GVCC → GPI	0.70	0.001	0.45~0.86
Indirect Effect			
GVCC → TI → GPI	0.17	0.02	0.05~0.30
Direct Effect			
GVCC → GPI	0.53	0.002	0.28~0.81

Abbreviations: BC, bias-corrected.

## Data Availability

Specific details regarding the data supporting the reported results can be obtained from the authors upon reasonable request.
